# Ingestive Behavior of Ovine Fed with Marandu Grass Silage Added with Naturally Dehydrated Brewery Residue

**DOI:** 10.1155/2016/5141674

**Published:** 2016-07-28

**Authors:** Daniele de Jesus Ferreira, Anderson de Moura Zanine, Rogério de Paula Lana, Alexandre Lima de Souza, Marinaldo Divino Ribeiro, Fagton Mattos Negrão, Wanderson José Rodrigues Castro, Henrique Nunes Parente, Luiz Juliano Valério Geron, Larissa Rodrigues de Azevedo Câmara

**Affiliations:** ^1^Department of Animal Science, Federal University of Maranhão, 65500-000 São Luís, MA, Brazil; ^2^Department of Animal Science, Federal University of Viçosa, 36570-977 Viçosa, MG, Brazil; ^3^Department of Animal Science, Federal University of Mato Grosso, 78735-901 Cuiabá, MT, Brazil; ^4^Department of Animal Science, Federal University of Goiás, 74690-900 Jataí, GO, Brazil; ^5^Department of Animal Science, State University of Mato Grosso, 78250-000 Sinop, MT, Brazil

## Abstract

The objective was to evaluate the ingestive behavior of ovine fed Marandu grass silage with dehydrated brewery residue added. The experiment had a completely randomized design with five treatments and four repetitions, with the treatments levels of inclusion being of 0, 10, 20, 30, and 40% natural matter of naturally dehydrated brewery residue for 36 hours to the marandu grass silage. 20 ovines were used and the experimental period was 21 days, 15 being for adaptation to diets. The use of brewery byproduct promoted quadratic effect (*P* < 0.05) for the consumption of dry matter with maximum point value estimated at adding 23.25% additive. Ingestion efficiency and rumination efficiency of dry matter (g DM/hour) were significant (*P* < 0.05), by quadratic behavior, and NDF ingestion and rumination efficiency showed crescent linear behavior. The DM and NDF consumption expressed in kg/meal and in minutes/kg were also significant (*P* < 0.05), showing quadratic behavior. Rumination activity expressed in g DM and NDF/piece was influenced (*P* < 0.05) by the adding of brewery residue in marandu grass silage in quadratic way, with maximum value estimated of 1.57 g DM/bolus chewed in inclusion of 24.72% additive in grass silage. The conclusion is that intermediary levels adding of 20 to 25% dehydrated brewery residue affects certain parameters of ingestive behavior.

## 1. Introduction

The need to intensify ovine meat production system in Mato Grosso, Brazil, has been attributed to the search for alternatives that enable better combinations of food and diet costs reduction. However, the physical and chemical properties of byproducts differ from foraging plants, which makes their degradation and passage by the gastrointestinal tract different and may affect the ingestive behavior, which is influenced by the physical structure and chemical composition of diets [1–3].

In such context, among the alternatives for yielding of industrial byproducts animal feeding shows great potential [4–6], since the increase in prices of vegetal supplementation used for animal feeding has aroused great interest by the yielding of nonconventional food. However, so that the correct use of such byproducts be performed, it is necessary to verify nutrition adequacy of such ingredients [2, 7]. This is important, for the correct formulation of diets for animals goes beyond fulfilling nutrition needs, including the quality of final product and fulfilling the expectations of consumers.

Ovine behavior varies according to the type of food. For animals kept in the field, long feeding periods are characteristics, according to the capacity of selection for each species [8]. However, for confined animals, the periods vary according to the energy level of supplied food. The nature of the diet also influences the ingestive behavior, with the rumination time probably being proportional to the level of the voluminous cellular wall [9].

However, knowledge about the animals' ingestive behavior that receives byproducts as part of their diet will contribute to food preparation, besides solving problem DM related to consumption diminishing. The presence of eventual antinutritional substances on food may alter time spent in feeding and, consequently, in rumination and rest periods [5]. Inclusion of brewery byproduct in preparation of diets may constitute an important feeding alternative, due to the high levels of gross protein, and, thus, increase energy outcome on diets [3].

Under such aspect, studies that assess the inclusion of brewery residue in adequate levels for ovines and its influence over the ovine ingestive behavior that receive diets containing such byproduct are still short, justifying the conducting of this research.

In this context, this experiment was conducted to evaluate ingestive behavior of ovine fed with marandu grass silage added with naturally dehydrated brewery residue.

## 2. Materials and Methods

### 2.1. The Study Site and Sampling Procedure

The experiment was conducted in the forage crops sector experimental area in the Department of Animal Science, Federal University of Mato Grosso, Rondonópolis, Brazil, at geographical coordinates 16°28′S and 50°34′W.

The climate is of Aw tropical type by Köppen classification, with well-defined wet and dry seasons; hot and humid summers; and cold and dry winters. The mean annual temperature is 27.5°C, oscillating between respective minimum and maximum means of 17°C and 38°C. The mean relative humidity of the air is 60% and the mean annual precipitation is 1240 mm.

The researched forage species was* Brachiaria brizantha* cv. Marandu. For the conduction of the experiment, uniformization harvest of the forage was performed in January 2011, with tractor attached harvester, to a height of approximately 5 cm of the ground. On the same day a maintenance fertilization was performed with 60 kg of nitrogen and potassium, in the form of urea and potassium chloride, respectively, and, after 60 days, the forage harvest was performed for the silage process.

Following that, the storage in silos was performed, with 200 liters of capacity; the silo was opened after 45 days. The experimental lining was entirely random, with five treatments and four repetitions, with the treatments levels of inclusion being of 0, 10, 20, 30 and 40% natural matter of naturally dehydrated brewery residue for 36 hours to the marandu grass silage to feed the sheep.

The evaluation period had a total duration of 21 days, with 15 days for adaptation to the diets and six for data collection. In the first phase, feed was provided* ad libitum* and consumption was measured daily, with the greatest mean consumption occurring on the third day of adaptation taken as a basis for providing during the collection phase. The silage was provided daily at 8:30 and 16:30 hours, for the duration of the experimental period. In addition to silage, the sheep were offered mineral salt supplement* ad libitum*, with its composition as follows: phosphorus = 80 g; calcium = 177 g; sulfur = 20 g; sodium = 40 mg; copper = 550 mg; iodine = 60 mg; selenium = 15 mg; manganese = 1200 mg; zinc = 3000 mg; and fluorine (max) = 800 mg.

The quantity of silage provided to each animal in the collection phase was 10% greater than the mean consumption observed in the preliminary phase, in order to enable leftovers. The consumption of silage was measured daily by measuring the weight difference between the feed offered and the leftover feed. Composite samples of provided and leftover feed was later placed in plastic wrap, identified, and stored in a freezer for laboratory analyses.

### 2.2. Chemical Composition and Digestibility of Marandu Grass Silage

When the silos opened, subsamples were collected of approximately 25 g for pH analysis, to which 100 mL water was added, and, after a two-hour rest, the pH assessment was performed using a potentiometer. To another 25 g subsample, 200 mL of a H_2_SO_4_ solution and 0.2 N were added, remaining at rest for 48 hours and then being filtered in a Whatman 54 type filter. This filtered byproduct was stored in a refrigerator for further n-ammoniacal analysis.

The buffering capacity analysis was determined in frozen samples, according to the technique described by [10]. For this purpose 15 to 20 g of fresh material was weighted, performing the maceration in a blender with 250 mL distilled water. The mix macerated material plus distilled water was titrated, firstly, for 3.0 pH with HCl 0.1 N to release bicarbonates and CO_2_ and, then, titrated for 6.0 pH with NaOH 0.1 N. The buffering capacity was expressed with equivalent alkali milligram required to change pH from 4.0 to 6.0 by 100 g dry matter, after correction for the titration value of 250 mL water.

The soluble carbohydrates were extracted by percolation with 80% ethanol, in the reaction with acid solution prepared with anthrone and in the following reading in spectrophotometer using the glucose solution for the standard curve preparation. The total digestible nutrients (TDN) were estimated according to [9], by the equation TDN (%) = Deg + (1.25 *∗* EE) − MM, where Deg is degradability; 1.25 is correction factor; EE is ethereal extract; MM is mineral matter.

Collection samples of the silage were dried in a forced air oven at 65°C for 48 hours and then ground in a Wiley mill, equipped with sieve mesh of 1 mm, according to the recommendations of [11].

To the tubes, 40 mL of McDougall solution (artificial saliva) and 10 mL innocuous rumen of animals grazing marandu grass were added.* In vitro* dry matter digestibility (IVDMD) was determined following the methods of Tilley and Terry [12], by incubating in a thermostatically controlled water circulating bath. The tubes were sealed with rubber corks containing bunsen valves (immediately after CO_2_ passage) and incubated for 48 hours in controlled temperature greenhouse, being agitated at least three to four times during fermentation. The second phase occurred after supernatant centrifugation and discarding. A pepsin solution (1 : 10.000) was added (50 mL) at 0.2% in each tube, agitated and placed in greenhouses at 39°C for 48 hours. After tubes washing, drying, and weighing, the calculations were performed according to the following: IVDMD = 100 × g sample DM − (g sample DM − g white DM)/g sample DM.

Ground samples were stored in polyethylene containers for further analysis of dry matter (DM), crude protein (CP), neutral detergent fiber (NDF), acid detergent fiber (ADF), hemicellulose (HEM), ethereal extract (EE), and crude ash (CA) according to methodology described by (AOAC, 2005).

The total digestible nutrient (TDN) values were estimated according to [9], by the following equation: TDN (%) = Deg + (1.25 *∗* EE) − CA, where Deg is degradability, 1.25 is correction factor, EE is ether extract, and CA is ash. The content of neutral detergent fiber was determined according to [9].

The chemical composition, pH, and N-NH_3_ of marandu grass silage added by different levels of dehydrated brewery residue are presented in Table 1.

### 2.3. Animals' Feeding Behavior

Animals' feeding behavior was determined in the last five days of the experimental period by quantifying the time intervals, for 24 hours [13]. In the recorded time spent in feeding, rumination, and resting, the visual observation of the animals for every 10 minutes is done by six trained observers, in rotation system, strategically placed in order to not bother the animals. During the same period, the counting of cud chews (MMnb, number/piece) was performed using a digital chronometer. To obtain chewing averages and time, cud observations were performed every 30 minutes, within 24 hours of evaluation.

The variables DM g and NDF/bolus were obtained by dividing individual average consumption of each fraction by the number of ruminated boluses per day (within 24 hours). The efficiency of feeding and rumination, expressed in g DM/hour and g NDF/hour, was obtained by dividing the average daily intake of DM and NDF in the total time spent feeding and/or rumination in 24 hours, respectively.

This and other variables, such as the number of ruminated boluses per day (NBR), time of total chewing (TMT), and the number of cud chews per day (MMND), were obtained through methodology described by [14]. During data collection, in the night observation, the environment was kept under artificial lighting.

The number of feeding periods, rumination, and rest were calculated by the number of activity sequences observed in the note sheet. The average daily duration of these activity periods was calculated dividing the total duration of each activity (feeding, rumination, and rest in min/day) by its respective number of described periods.

### 2.4. Statistical Analysis

The remaining data were statistically analyzed: feeding behavior, the choice being based on the significance of the regression parameters, tested by Tukey (*P* < 0.05), and coefficients of determination models. Statistical analyses were performed using the statistic package [15].

## 3. Results and Discussion

### 3.1. Ingestive Behavior of Sheep

**Figure 1:**
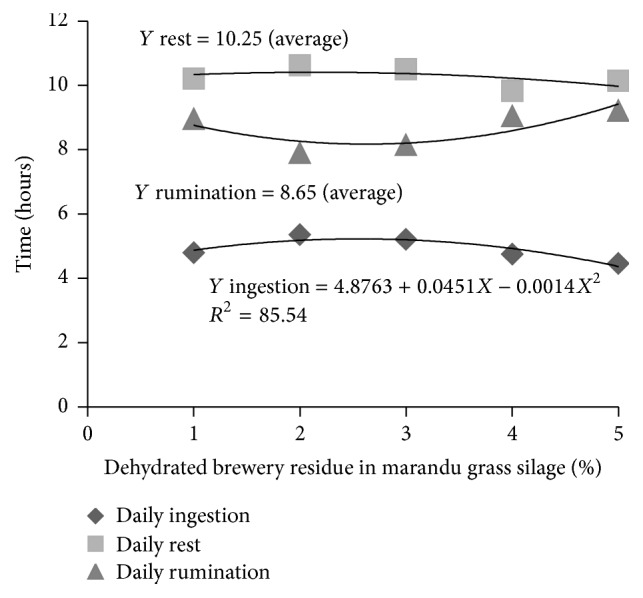
Ingestion time, rest, and rumination in hours, during the daily period (24 hours) of ovine fed with marandu grass silage.

Taking into account Figure 1, in which the times of ingestion, rumination, and day time rest are presented, which means the values of the sum of the night period and the day period, in a total of 24 hours of observation, one could verify if there were statistic differences for the daily ingestion time (*P* < 0.05), with quadratic effect, with the maximum value being estimated in 5.56 hours for the level of 16.10% dehydrated brewery residue, a result very close to the day ingestion time presented in Figure 1, which was expected, as longer ingestion periods occurred during the day. We verified the intermediate inclusion levels of dehydrated brewery residue, in the production of marandu grass silage, showed higher ingestion times.

There was no effect (*P* > 0.05) over the rumination time, which showed average values of 8.95; 7.89; 8.14; 9.03; and 9.20 hours, respectively. Similar behavior was verified for the daily rest time with average value of 10.25 hours.

The use of brewery byproduct promoted quadratic effect (*P* < 0.05) over the consumption of dry matter with maximum value point estimated at 23.25% additive (Table 4). The initial increase of consumption of dry matter could be explained, in part, by the increase of dry matter level in silage. By adding dehydrated brewery residue, with 90% dry matter in silage, part of the grass humidity was absorbed by the residue, improving fermentation quality (Table 3), with average pH and N-ammoniacal of 4.13 and 6.16%, respectively, in the silage with additive.

An indicator of this improvement was observed in the silage containing dehydrated brewery residue, which showed appropriate and pleasant odor and color, although silage only with marandu grass had shown good fermentation, due to the fact that it was ensiled with approximately 25% dry matter, with recommended average level being of 30 to 35% dry matter, according to [10].

References [6, 7] show that the use of industrial byproducts requires special attention, mostly to what concerns stimulation of rumination function, for sources of alternative diet fiber deserve special attention, mainly because grounding may reduce particle size and, thus, can result in metabolic alterations. By consequence, although the 40% level of dehydrated brewery residue is a high percentage in grass silage, increasing reduced particles, thus, an increase was expected in consumption of dry matter, stimulated by higher rumen passage rate, but such fact was not observed, for the consumption showed quadratic behavior.

Crescent linear behavior was observed (*P* < 0.05) of the regression equation for the consumption of fiber in neutral detergent (NDF) in 24-hour assessment (Table 2). There was an increase of 0.005% in NDF consumption for each 1% dehydrated brewery residue added. The maximum inclusion level (40%) estimated by the equation was 0.488 g, which means the ovine consumed 0.288 kg more than in control treatment (0.260 kg). Reference [16], assessing elephant grass silage with different proportions of dehydrated passion fruit husk, in lamb diet, observed that the NDF consumption in kg/day increased from 0.004% for each 1% of passion fruit husk included in grass silage, values close to the ones observed in the present study.

However, the number of ruminated boluses per day (number/day) showed decreasing linear behavior (*P* < 0.05), so the maximum value was 653.08 boluses for control silage and for the highest dehydrated brewery residue level was 595.8  ruminated boluses/seconds. This could indicate that such level might have undesired influence in rumen, reducing pH since the saliva stimulation is lower, thus influencing consumption (Table 2). However, ingestive behavior evaluation, by the average time spent in chewing per bolus (seg) showed quadratic behavior (*P* < 0.05), and the maximum value was 51.15  ruminated boluses/second for the level of 27.85% dehydrated brewery residue (Table 2).

Such behavior highlights that the reduction of cud per day, with dehydrated brewery residue added, was compensated by the increase of chew time per bolus (Table 4), which explains the raise in the number of cud chews per bolus, which behaved quadratically (*P* < 0.05), in which it reached the maximum point of 74.19  boluses chewed in the level of inclusion of 21.20% dehydrated brewery residue (number/bolus) (Table 3), behavior similar to the one observed for the chew time per bolus (number/day).

Reference [17] described that the chewing activity stimulates saliva secretion and present buffers (bicarbonates and phosphates), with neutralization of the acids produced by the fermentation of cud's organic matter.

On the other hand, the total chews number showed quadratic behavior (*P* < 0.05), with maximum value of 23.405 chews per day for the level of 20.44% which could be related to the reduction of the number of ruminated boluses per day (Table 2).

Reference [18] studying the ingestive behavior of caprine and ovine fed with hay and/or cassava observed that there was significant difference for cud chewing time, especially for the animals that received hay, for they spent more time chewing than those that received silage. The authors recorded that, regardless of the treatment, caprine chewed the cud more than ovine, due to the fact that caprine have a higher capacity to take ingested food, when of worst quality, which is enabled by the better rumen environment and urea recycling, compared to ovine.

Ingestion efficiency (g DM/hour) was significant (*P* < 0.05), with quadratic behavior (Table 3), as the equation showed, for the 0, 20, and 40% levels of dehydrated brewery residue values of 175, 270, 182,09, and 187,42 g DM/hour, respectively, and NDF ingestion efficiency (g NDF/hour) showed crescent linear behavior, with values of 55,39, 95,58, and 135,77 g DM/hour, respectively, for these levels.

Thus, there was efficiency increase of NDF ingestion of 2% for each 1% dehydrated brewery residue included in grass silage. It is noteworthy that, in this study, the DM and NDF consumptions (kg/day) were reflexes of the added silage and showed average values of, respectively, 0.835 and 0.375 kg, which justifies the results obtained for the ingestion efficiencies (g DM/hour), which are directly related to consumption expressed in g/day.

For the rumination efficiency of dry matter (g DM/hour), quadratic behavior was observed for the equation (*P* < 0.05), the maximum estimated value being of 107.93 g DM/hour, with the level of 21.83% dehydrated brewery residue added in marandu grass silage (Table 3). Efficiency of fiber in neutral detergent rumination (g NDF/hour) had crescent linear response (*P* < 0.05), in which each 1% inclusion of dehydrated brewery residue in marandu grass silage promoted increase of 0.59%.

According to [9], the fiber level and the physical shape of diet are the main factors that affect rumination time. Since the diets show reduced NDF levels due to the adding of dehydrated brewery residue, rumination efficiency was affected, for, according to [5–19], the number of periods of rumination increases according to the level of fiber in diet, which reflects the need of processing rumen digestion to increase digestive efficiency.

The ingestion period (number/day) was significant (*P* < 0.05) showing quadratic effect with maximum estimated value of 31.69/day, with the inclusion level of 20.03% of dehydrated brewery residue (Table 3).

DM and NDF consumption expressed in kg/meal and in minutes/kg were also significant (*P* < 0.05), showing quadratic behavior (Table 4). Significant differences in those variables are expected, since the ingestion time, the number of daily meals, and the consumption of DM and NDF were influenced in a quadratic form and/or linear by the adding of dehydrated brewery residue.

The time spent per period (min) was not significant (*P* > 0.05) for the rumination and rest periods (min) and, for the ingestion period (min), there was quadratic behavior (*P* < 0.05), which reflected the similarity in ingestion, rumination, and rest times (hours/day) and the number of periods for each activity (number/day) between the tested silage, indicating that the use of dehydrated brewery residue in marandu grass production does not affect discretion of the time series in ovine in the rumination and rest conditions (Table 4).

The results were concordant with the ones from [20], assessing the levels of 0, 10, 20, and 30% cacao bran in ovine's diet, in which the authors also did not observe differences in the period spent for rumination and rest, in minutes or number per day.

The rumination activity, expressed in g DM and NDF/bolus, was influenced (*P* < 0.05) by the adding of brewery residue in marandu grass silage in a quadratic form (Table 5), with maximum estimated level of 1.57 g DM/bolus, in the inclusion of 24.72% of the additive in grass silage. Similar behavior was observed for the time spent in rumination (NDF/bolus) (Table 5). The rumination activity, expressed in min/kg DM and NDF, was also influenced (*P* < 0.05) by adding brewery residue in marandu grass silage quadratically (Table 5), with minimum estimated value of 570.38 min/kg DM ruminated, in the inclusion of 21.69% brewery residue. Similar behavior was recorded for rumination activity, expressed in min/kg NDF.

Such behaviors can be influenced by the NDF levels in silage, which varied with the inclusion of dehydrated brewery residue, with the maximum difference between the levels of this fraction (13.13%) in silage with 0 and 40% additive, which might have been enough to provoke alterations in the rumination activities. A factor that might have favored the presence of effect over rumination is the small size of the particles of the dehydrated brewery residue, similar to concentrated food such as grounded corn and soy bran [21, 22].

Total chewing time, in min/kg of DM and NDF, was influenced quadratically (*P* < 0.05) by the levels of brewery residue in marandu grass silage (Table 5), due to the similarity between times spent in ingestion and rumination, since the total time is obtained by the sum of times spent in ingestion and rumination within 24 hours.

## 4. Conclusions

Use of dehydrated brewery residue in the process of silage of marandu grass affects some parameters of ingestive behavior, such as ingestion time, dry matter consumption, consumption of fiber in neutral detergent, number of ruminated boluses per day, ingestion efficiency indicating the use of intermediate level of 20 to 25%.

## Figures and Tables

**Table 1:** Chemical composition, pH, and N-NH_3_ of marandu grass silage with dehydrated brewery residue added.

Variable	DBR^*∗*^	Dehydrated brewery residue (%)				
		0	10	20	30	40
pH	—	4.28	4.19	4.13	4.12	4.09
N-NH_3_^1^	—	7.61	7.03	6.45	5.89	5.29
Dry matter^1^	89.96	24.13	27.91	31.02	39.5	43.9
Nitrogen^2^	3.89	1.15	1.80	2.02	2.16	2.35
Soluble carboydrates^2^	—	4.06	4.09	4.12	4.15	4.19
Ether extract	6,52	3.52	5.08	5.19	5.29	5.42
Total digestible nutrient^2^	—	33.27	39.06	44.85	50.60	56.40
Neutral detergent fiber^2^	60.75	73.63	64.06	61.25	61.60	60.50
Acid detergent fiber^2^	30.09	29,45	33.28	30.96	32.30	34.50
Hemicellulose^2^	30.66	44.18	33.78	30.29	29.30	26.00
Lignin^2^	5,48	6.89	6.48	6.40	6.32	6.18
Crude ash^2^	8.89	6.31	7.13	7.02	6.52	6.55
*In vitro* dry matter digestibility^2^	64.35	60.93	64.37	65.10	65.60	66.10

^1^Percentage.

^2^Percentage of dry matter.

^*∗*^Dehydrated brewery residue.

**Table 2:** Dry matter (kg/day) and neutral detergent fiber (NDF/kg/day) consumption in 24 hours, number of ruminated boluses per day (number of boluses per day), average time (sec) spent in chewing per bolus (boluses per sec), and total time chew (min/day) of ovine fed with marandu grass silage.

Variable	Dehydrated brewery residue (%)					Equation regression^*∗∗*^	CV (%)	*R* ^2^ (%)
	0	10	20	30	40			
Dry matter	0.744	0.820	0.864	0.875	0.852	*Y* = 0.744 + 0.0093*X* − 0.0002*X* ^2^	8.13	86.20
NDF	0.282	0.302	0.332	0.480	0.482	*Y* = 0.26 + 0.0057*X*	20.31	87.32
Number of boluses per day	652.6	638.1	623.6	609.1	594.6	*Y* = 653.08 − 1.450*X*	11.01	81.10
Chews	44.63	49.48	52.20	52.81	51.29	*Y* = 42.931 + 0.590*X* − 0.0106*X* ^2^	8.02	81.76
Total time chew	18966	22246	23402	22434	19342	*Y* = 18967.7 + 434.2*X* − 10.62*X* ^2^	15.36	86.66

^*∗∗*^Significant by Tukey test at 0.5% probability.

**Table 3:** Feeding efficiency (g DM and NDF/hour), rumination efficiency (g DM and NDF/hour), cud chews, and number of meal periods, rumination, and rest (number/day) of ovine fed with marandu grass silage.

Variable	Dehydrated brewery residue (%)					Equation regression^*∗∗*^	CV (%)	*R* ^2^ (%)
	0	10	20	30	40			
Ingestion efficiency (g DM and NDF/hour)								
DM	162.0	169.3	176.6	184.0	185.3	*Y* = 175.27 + 0.371*X* − 0.0015*X* ^2^	17.02	89.14
NDF	65.38	59.49	65.55	103.1	113.9	*Y* = 55.398 + 2.0093*X*	20.89	90.55

Rumination efficiency (g DM and NDF/hours)								
DM	85.23	101.2	107.7	104.7	92.26	*Y* = 85.332 + 2.070*X* − 0.0474*X* ^2^	12.98	96.72
NDF	32.15	38.12	44.09	50.05	56.02	*Y* = 32.152 + 0.596*X*	20.46	86.63

Cud chewing								
Hours (day)	13.47	13.46	13.40	13.59	13.94	14.172	—	—
Number of boluses per day	67.62	72.35	74.15	73.02	68.97	*Y* = 67.6298 + 0.6191*X* − 0.014*X* ^2^	10.88	86.35
Number/day	48535	43739	41624	42191	45439	*Y* = 48534.19 − 13.69*X* + 13.40*X* ^2^	16.32	74.79

Ingestion period, rumination, and rest (number/day)								
Ingestion	27.92	30.75	31.70	30.77	27.97	*Y* = 27.921 + 0,3766*X* − 0.0094*X* ^2^	17.08	85.13
Rumination	52.95	49.43	48.72	50.81	55.81	51.54	—	—
Rest	62.12	62.71	62.22	60.66	58.02	61.14	—	—

^*∗∗*^Significant by Tukey test at 0.5% probability.

**Table 4:** Neutral detergent fiber (min/kg) and time spent per ingestion period, rumination, and rest (min) of ovine fed with marandu grass silage.

Variable	Dehydrated brewery residue (%)					Equation regression^*∗∗*^	CV (%)	*R* ^2^ (%)
	0	10	20	30	40			
DM and NDF consumption/meal (kg)								
DM	0.027	0.028	0.029	0.030	0.032	*Y* = 0.027 + 0.00017*X* − 0.0001*X* ^2^	20.63	91.84
NDF	0.011	0.010	0.012	0.015	0.021	*Y* = 0.0116 + 0.00017*X* − 0.00001*X* ^2^	22.45	74.93

DM and NDF consumption (min/kg)								
DM	392.9	383.2	365.9	341.2	309.1	*Y* = 392.92 + 0.6046*X* − 0.0373*X* ^2^	17.46	79.81
NDF	1119	989,2	9859	729,1	599,1	*Y* = 1084.64 − 6.0861*X* − 0.1721*X* ^2^	22.33	88.37

Time spent per ingestion period, rumination, and rest (min)								
Ingestion	10.64	10.28	9.97	9.71	9.51	*Y* = 10.6399 − 0.0389*X* + 0.0003*X* ^2^	12.02	91.74
Rumination	9.98	10.04	10.10	10.17	10.23	10.10	—	—
Rest	11.97	12.92	13.03	12.29	10.71	9.78	—	—

^*∗∗*^Significant by Tukey test at 0.5% probability.

**Table 5:** Time spent in rumination (g DM and NDF/bolus), rumination (min/kg DM and NDF), and total chewing (min/kg DM and NDF) of ovine fed with marandu grass silage.

Variable	Dehydrated brewery residue level (%)					Equation regression^*∗∗*^	CV (%)	*R* ^2^ (%)
	0	10	20	30	40			
Rumination (g DM and NDF/cud)								
DM	1.04	1.39	1.55	1.52	1.30	*Y* = 1.0399 + 0.0445*X* − 0.0009*X* ^2^	13.57	92.97
NDF	0.376	0.531	0.649	0.733	0.781	*Y* = 0.377 + 0.0171*X* − 0.00017*X* ^2^	23.62	96.36

Rumination (min/kg DM and NDF)								
DM	707.3	610.4	571.5	590.7	667.8	*Y* = 706.99 − 12.590*X* + 0.2901*X* ^2^	12.75	88.34
NDF	1941	1628	1397	1249	1184	*Y* = 1941.14 − 35.414*X* + 0.142*X* ^2^	20.32	95.81

Total chewing (min/kg DM and NDF)								
DM	999.9	921.4	883.5	886.4	929.9	*Y* = 999.99 − 9.887*X* + 0.2034*X* ^2^	11.66	91.59
NDF	2560	2252	2019	1863	1782	*Y* = 2561.27 − 34.676*X* + 0.389*X* ^2^	13.38	94.80

^*∗∗*^Significant by Tukey test at 0.5% probability.
